# Supplementary figures and images for: Impact of the Duration of Postoperative Antibiotics on the Prognosis of Patients with Infective Endocarditis
Source: Antibiotics (Basel). 2023 Jan 15;12(1):173. doi: 10.3390/antibiotics12010173 (PMC9854446; doi:10.3390/antibiotics12010173)

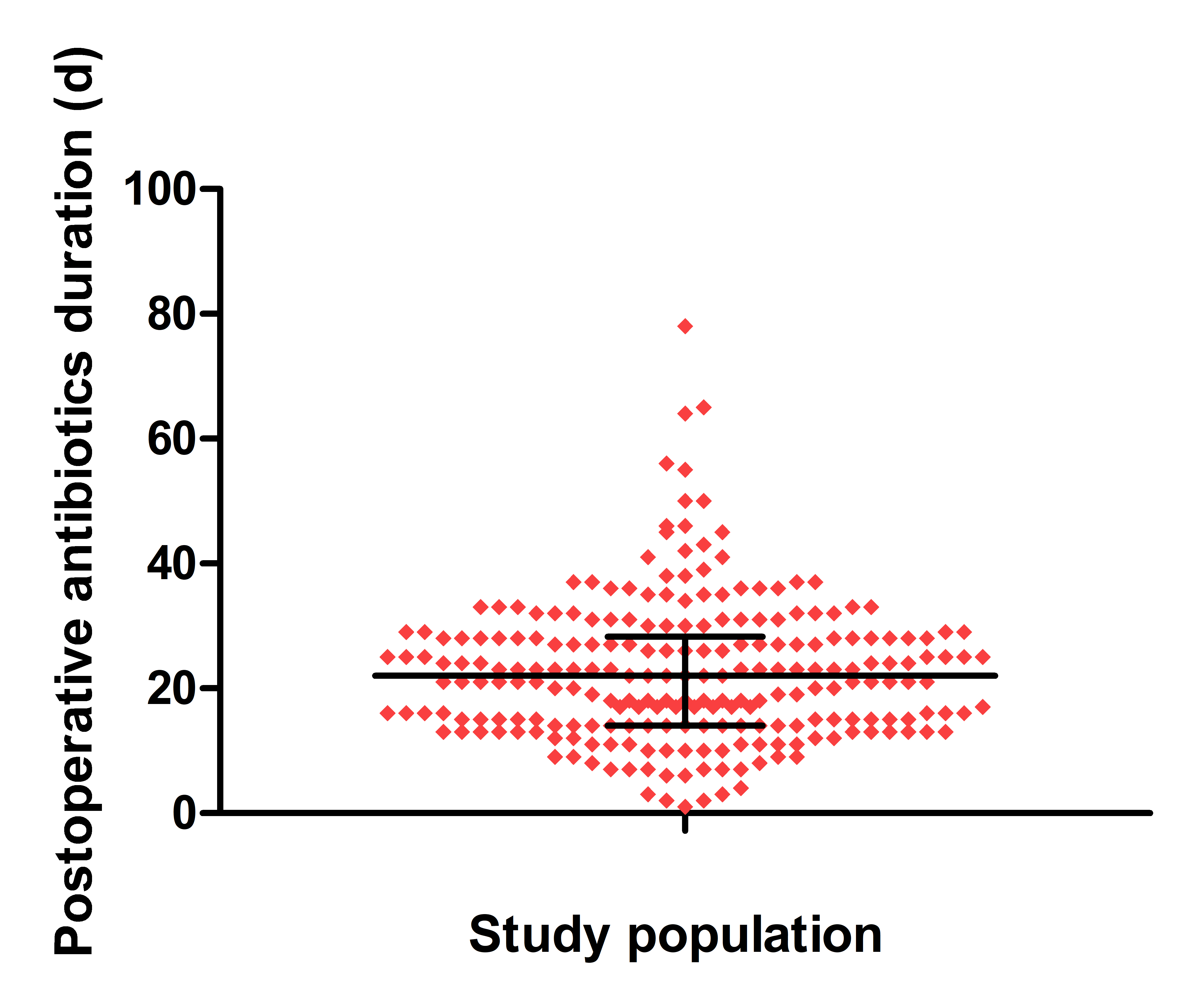

Supplement: Supplementary file 1 [file antibiotics-12-00173-s001.zip › antibiotics-2054127-supplementary.jpg]
